# Outbreak of neuropathogenic equid herpesvirus 1 causing abortions in Yili horses of Zhaosu, North Xinjiang, China

**DOI:** 10.1186/s12917-022-03171-1

**Published:** 2022-03-01

**Authors:** Panpan Tong, Ruli Duan, Nuerlan Palidan, Haifeng Deng, Liya Duan, Meiling Ren, Xiaozhen Song, Chenyang Jia, Shuyao Tian, Enhui Yang, Ling Kuang, Jinxin Xie

**Affiliations:** 1 Laboratory of Animal Etiology and Epidemiology, College of Veterinary Medicine, Xinjiang Agricultural University, Urumqi, Xinjiang China; 2 Zhaosu Horse Barn in Yili, Zhaosu, China; 3 Center of Animal Disease Control and Prevention, Acheng District, Harbin, Heilongjiang China; 4 Cisen Pharmaceutical Co., Ltd, Jining, Shandong China; 5 Tiankang biological Co., Ltd, Urumqi, Xinjiang, China

**Keywords:** Yili horses, Abortion storm, Equid herpesvirus 1, ORF33 gene, ORF30 gene, Neuropathogenicity

## Abstract

**Background:**

EHV-1 is one of the most serious viral pathogens that frequently cause abortion in horses around the world. However, so far, relatively little information is available on EHV-1 infections as they occur in China. In January 2021, during an abortion storm which occurred in Yili horses at the Chinese State Studs of Zhaosu (North Xinjiang, China), 43 out of 800 pregnant mares aborted.

**Results:**

PCR detection revealed the presence of EHV-1 in all samples as the possible cause of all abortions, although EHV-4, EHV-2 and EHV-5 were also found to circulate in the aborted fetuses. Furthermore, the partial ORF33 sequences of the 43 EHV-1 shared 99.3–100% and 99.0–100% similarity in nucleotide and amino acid sequences respectively. These sequences not only indicated a highly conserved region but also allowed the strains to group into six clusters. In addition, based on the predicted ORF30 nucleotide sequence, it was found that all the strains carried a guanine at the 2254 nucleotide position (aspartic acid at position 752 of the viral DNA polymerase) and were, therefore, identified as neuropathogenic strains.

**Conclusion:**

This study is the first one that establishes EHV-1 as the cause of abortions in Yili horses, of China. Further characterization of the ORF30 sequences revealed that all the EHV-1 strains from the study carried the neuropathogenic genotype. Totally, neuropathogenic EHV-1 infection in China’s horse population should be concerned although the virus only detected in Yili horse abortions.

**Supplementary Information:**

The online version contains supplementary material available at 10.1186/s12917-022-03171-1.

## Background

Equid herpesvirus 1 (EHV-1), classified within the family *Herpesviridae*, the subfamily *Alphaherpesvirinae* and the genus *Varicellovirus* [1], is one of the most serious viral pathogens of the horse population as it is frequently associated with abortion, respiratory problems as well as both ocular and neurological diseases in horses. As such, it can have a significant impact not only on equine health but also on the overall equine industry around the world [2–7].

EHV-1 possesses a 150 kbp double-stranded DNA genome which encodes up to 80 open reading frames (ORFs) [8–10]. Among these, ORF33, encoding the envelope glycoprotein B (gB), possesses a conserved region that is frequently targeted for diagnostic PCR and phylogenetic analyses [11]. Similarly, ORF30 encodes the viral DNA polymerase which is strongly but not exclusively associated with both non-neuropathogenic or neuropathogenic EHV-1 depending on whether an adenine (A) or guanine (G) is present at position 2254 respectively. This, in turn, corresponds to an asparagine (N) or aspartic acid (D) at amino acid position 752 of the viral DNA polymerase, respectively [11–24].

In January 2021, 43 Yili horses from the Chinese State Studs of Zhaosu (Northern Xinjiang, China) aborted without any additional clinical signs. These were part of a population of 820 horses of which 20 were Thoroughbred stallions (> eight years of age) and 800 were mares (> four years of age) at approximately six months of gestation. In fact, horses of this farm had no vaccination scheduled, and they encountered abortion storms every year. In order to establish viral pathogens as the possible cause of these abortions, lung tissues of all aborted fetuses were analyzed by PCR to check for the presence of EHV-1, equid herpesvirus 4 (EHV-4), equid herpesvirus 2 (EHV-2) and equid herpesvirus 5 (EHV-5). In addition, the ORF30 and ORF33 genes of EHV-1 strains detected in the samples of aborted fetuses were further characterized. This study is the first to have been carried out on viruses responsible for abortion in the Yili horses of Xinjiang, one of the major horse-producing regions in China. In fact, in a general way, this work also represents the first to investigate viruses related to horse abortion in the country.

## Results

### EHV detection

The PCR results confirmed the presence of EHV-1, EHV-4, EHV-2 and EHV-5 in the lung tissues of aborted fetuses, with the percentage of positive cases for each virus being 100% (43/43), 2.3% (1/43), 7% (3/43) and 16.3% (7/43), respectively. Coinfection with EHV-1, EHV-4 and EHV-5 was detected in one aborted sample, coinfection with EHV-1, EHV-2, and EHV-5 was detected in three aborted samples, and coinfection with EHV-1 and EHV-5 was detected in three aborted samples. These results, therefore, suggested that EHV-1 could have been the cause of the abortion storm in the Yili horses.

### ORF33 sequences comparison and phylogenetic analysis

**Fig. 1:**
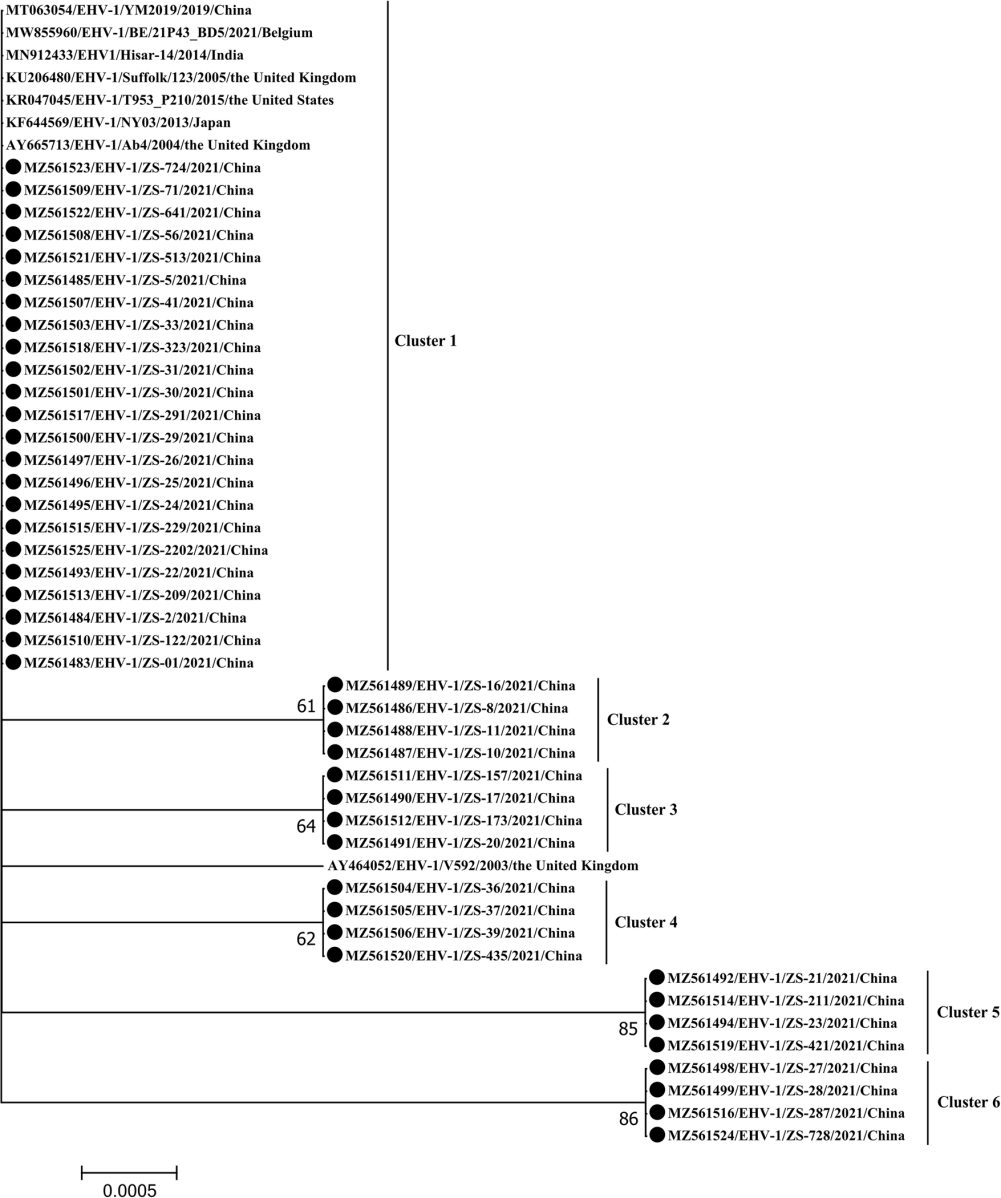
Phylogenic network representation based on EHV-1 ORF33 gene sequences using the maximum-likelihood method based on the Tamura–Nei model (MEGA7). The black circles indicate the EHV-1 strains identified in this study

Comparison of the partial ORF33 (592 nt) sequences from the 43 EHV-1 positive samples revealed a nucleotide and amino acid similarity level of 99.3–100% and 99.0–100%, respectively (accession nos: MZ561483-MZ561525), thus indicating that the partial ORF33 genes of China’s EHV-1 had a high sequence similarity. In addition, these sequences were also compared with those of EHV-1 reference strains identified in the United Kingdom (V592: accession no. AY464052, Ab4: accession no. AY665713, and Suffolk/123/2005: accession no. KU206480), in Japan (NY03: accession no. KF644569), in the United States (T953_P210/2015: accession no. KR047045), in India (Hisar-14/2014: accession no. MN912433), in China (YM2019: accession no. MT063054) and in Belgium (BE/21P43_BD5: accession no. MW855960). In this case, the results indicated a percentage similarity level of 99.5–100% and 99.0–100% for DNA and amino acid sequences (Table 1), respectively. Finally, a phylogenic network representation which was constructed based on those partial ORF33 nucleotide sequences revealed that EHV-1 strains in this study were clustered into six different clusters (Fig. 1).

**Table 1:** Nucleotide (upper right) and amino acid (bottom left) similarity of the ORF33 sequence between EHV-1 identified in this study and EHV-1 reference strains

	43 EHV-1 detected in this study	EHV-1 Ab4	EHV-1 NY03	EHV-1 T953_P210	EHV-1 Suffolk/123	EHV-1 Hisar-14	EHV-1 YM2019	EHV-1 BE/21P43_BD5	EHV-1 V592
**43 EHV-1 detected** **in this study**		99.7–100	99.7–100	99.7–100	99.7–100	99.7–100	99.7–100	99.5–99.8	99.5–99.8
**EHV-1** **Ab4**	99.5–100		100	99.9	99.9	99.8	100	99.9	99.9
**EHV-1** **NY03**	99.5–100	100		99.9	99.9	99.8	100	99.9	99.9
**EHV-1** **T953_P210**	99.5–100	99.7	99.7		99.9	99.9	99.9	99.9	99.9
**EHV-1** **Suffolk/123**	99.5–100	99.9	99.9	99.8		99.8	99.9	99.9	99.9
**EHV-1** **Hisar-14**	99.5–100	99.8	99.8	99.9	99.9		99.8	99.8	99.9
**EHV-1** **YM2019**	99.5–100	100	100	99.7	99.9	99.8		99.9	99.9
**EHV-1** **BE/21P43_BD5**	99.0–99.5	99.7	99.7	99.8	99.8	99.9	99.7		99.9
**EHV-1** **V592**	99.5–100	99.8	99.8	99.9	99.9	100	99.8	99.9

### ORF30 sequences analysis

**Fig. 2:**
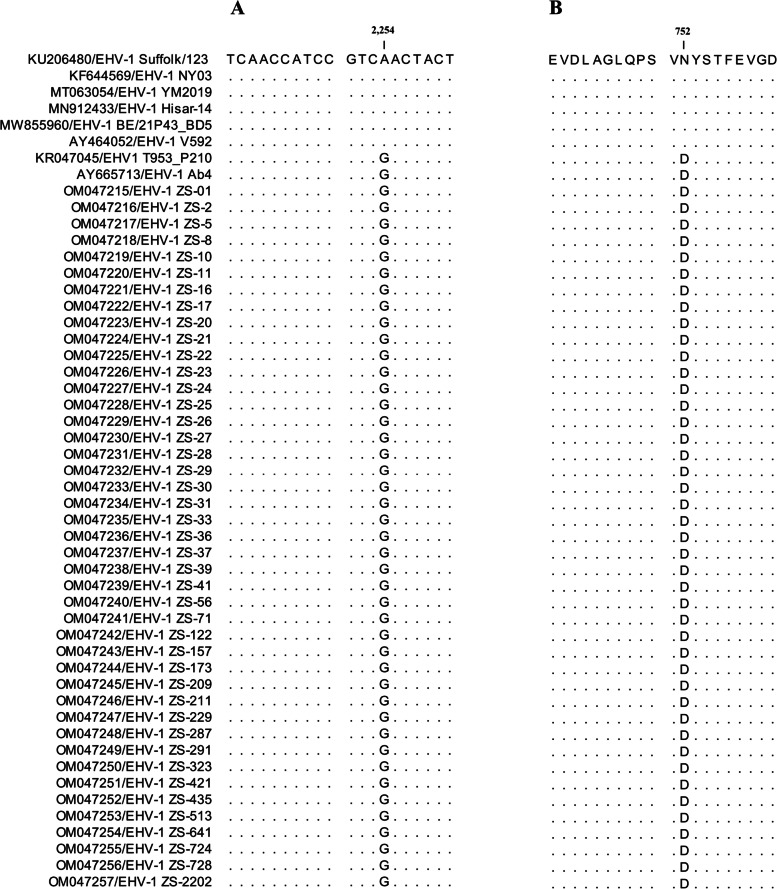
Analysis of ORF30 gene sequence. **A** Shows the presence of G2254 polymorphism in all the EHV-1 strains from this study. **B** Shows the presence of the D752 polymorphism in all the EHV-1 strains from this study. Dots indicate sequence identity in the alignment when compared to the EHV-1 Suffolk/123 isolate (CLC Sequence Viewer 8)

To investigate whether the 43 EHV-1 strains detected in this study were neuropathogenic or non-neuropathogenic in nature, partial PCR amplification of the ORF30 gene of EHV-1 (559 nt) was performed using specific primers (Table S1). In this case, the results showed that the partial ORF30 sequences of the 43 EHV-1 positive samples had 100% identity in their nucleotide and amino acid sequences (accession nos: OM047215-OM047257), hence indicating a high genetic conservation. Furthermore, in a similar way to the neuropathogenic EHV-1 referenced strains Ab4 and T953_P210, the ORF30 sequences of the 43 EHV1 strains of the study carried a guanidine (G) at position 2254 (D in position 752 of the viral DNA polymerase) (Fig. 2), and the detected strains were, therefore, identified as neuropathogenic EHV-1.

## Discussion

Horses represent a natural host for alphaherpesviruses (EHV-1 and EHV-4), and gammaherpesviruses (EHV-2 and EHV-5) [2, 16], with EHV-1 being the most common viral cause of abortion storms in horses.

Usually, these four EHVs occur together in aborted samples [2, 16] and hence, the findings of this study are in agreement with published reports regarding the detection of EHV-2, EHV-4, and EHV-5 as co-infections in aborted fetuses [2]. However, although coinfections with EHV-2 (accession nos: OK362329-OK362331), EHV-4 (accession no: OK362332), EHV-5 (accession nos: OL440383-OL440384) alongside EHV-1 were detected, only the latter was identified in all the fetal samples of the unvaccinated Yili horses. Therefore, the results suggested that EHV-1 could have been the main cause of the abortion storm in the Yili horse population included in this study. In fact, this is supported by previous results which revealed that EHV-1 was frequently involved in outbreaks that led to horse abortions around the world [2, 11–24]. Regarding the other viruses, EHV-4 is known to provoke sporadic abortions, but it has not yet been demonstrated whether EHV-2 and EHV-5 are also a cause for concern [16, 25, 26]. Given that these three viruses were detected in the EHV-1 positive aborted samples, there is the need to isolate them in order to demonstrate whether they can be the cause of abortion in Yili horses. In this context, a previous study actually noted that these three viruses circulated in thoroughbred foals with respiratory diseases in North Xinjiang [27], but here, the detection of these strains is being reported for the first time in abortion cases of horses in North Xinjiang. Therefore, based on the results, it can be concluded that EHV-2, EHV-4 and EHV-5 might contribute to abortions and respiratory diseases in the horses of Xinjiang, China.

In a similar way to one previous study, some variations in ORF33 genes of the EHV-1 viruses were also detected in the aborted samples [11]. Indeed, the partial ORF33 (592 nt) sequences from the 43 EHV-1 shared 99.3–100% nt identity, and clustered into six groups, thereby indicating genetic diversity.

Previous studies have shown that the nucleotide at position 2254 of ORF30 was strongly associated with the occurrence of EHV-1 neurological diseases [11–24]. For instance, most of the viruses recovered from abortion cases worldwide contained adenine (A) at this position (asparagine (N) at position 752 of the protein) and belonged to non-neuropathogenic strains (associated with non-neurological conditions) [11–24]. Similarly, as reported by Smith et al. (2010), G2254 strains have been isolated less frequently than A2254 ones in abortion cases [15]. However, in this study, the ORF30 sequences of 43 EHV-1 detected in the present study carried a G at position 2254 (D in position 752) (Fig. 2). Hence, the data provided evidence that neuropathogenic EHV-1 could have been responsible for the series of abortions in Yili horses. It should nevertheless be noted that comparative sequence analysis also showed similarity to EHV-1 YM2019 (accession no. MT063054) which, according to GenBank information, is non-neuropathogenic and was identified in aborted samples of Przewalski’s horses. As such, the results indicated that both non-neuropathogenic and neuropathogenic EHV-1 strains could be circulating in the horse population of Xinjiang, China. Indeed, while non-neuropathogenic EHV-1 had been detected from some cases of neurological diseases, in most cases, neuropathogenic EHV-1 was associated with the development of neurological diseases, higher levels of viraemia as well as longer disease duration. For instance, in Argentina, the United Kingdom and the United States, approximately 8.7–50% of neuropathogenic EHV-1, identified from abortion cases, were associated with neurological disease [12, 14]. These data clearly supported the results regarding the presence of neuropathogenic EHV-1 in Yili horses despite the absence of neurological signs.

Several published studies showed that only non-neuropathogenic EHV-1 are isolated in Brazil, Turkey and Poland [13, 17, 18]. Neuropathogenic EHV-1 has a low prevalence in Japan (2.7%), the United States (10.8–19.4%), Argentina (7.4%), France (24%), and Germany (10.6%) [4, 14, 15, 19–21], but a high prevalence in Italy (90%), Uruguay (92.3%) and Ethiopia (98.9%) [11, 22, 23]. However, over the past few decades, two investigations conducted in Italy and the United States indicated that the prevalence of neuropathogenic EHV-1 was on the rise. In addition, even though vaccination could reduce virus spreading, one study in Germany reported that vaccination against EHV-1 was not always successful at preventing the spread of the neuropathogenic genotype [24], with another study in Italy confirming that the spread of neuropathogenic EHV-1 strains were could be observed in both vaccinated mares as well as unvaccinated horses [11]. Thus, it is not surprising that all EHV-1 detected in aborted samples from unvaccinated Yili horses, could have been neuropathogenic EHV-1.

Furthermore, two recent studies in France and the United States identified a new EHV-1 DNA polymerase (ORF30) genotype. This was followed by the isolation of a C2254/H752 which was then associated with both respiratory and neurological clinical signs [28, 29]. Further study will investigate the presence of this new EHV-1 genotype in China’s horses.

## Conclusion

In summary, EHV-1, EHV-4, EHV-5 and EHV-2 were detected from samples of aborted fetuses, and as such, it was shown that EHV-1 could have been the cause of the abortion storm. Further characterization of the EHV-1 provided evidence that the neuropathogenic form of EHV-1 could have been involved. It is expected that this study will create awareness about the role of neuropathogenic EHV-1 in horse abortions in China, and promote the development of vaccines against the virus.

## Methods

### Sample collection

In January 2021, 43 out of 800 pregnant Yili mares from the Chinese State Studs of Zhaosu (Northern Xinjiang, China) aborted. Lung tissues of each aborted fetus were, therefore, transferred to a tube containing 10 mL of phosphate buffer for storage at − 80 °C until required for analysis.

### Viral nucleic acids extraction and sequencing

Lung tissue samples were ground and after centrifugation, viral nucleic acids were extracted from 200 μL of the supernatant (Geneaid Biotech Co.) by following the manufacturer’s instructions. The presence of EHV-1 (592 nt), EHV-2 (716 nt), EHV-4 (587 nt) and EHV-5 (881 nt) DNA in these nucleic acid samples was subsequently detected by PCR by targeting partial ORF33 (EHV-1 or EHV-4) or ORF8 (EHV-5 or EHV-2) genes (encoding the gB). For this purpose, primers were designed using publicly-available sequences in GenBank (accession nos: AY464052, LC075585, MK904567 and KC715730, Table S1) while the PCR itself was carried out using the TIANSeq HiFi Amplification Mix (Tiangen Biotech) with the following PCR conditions: an initial denaturation at 94 °C for 2 min, followed by 35 cycles, each with a denaturation step at 98 °C for 10 s, as well as both an annealing and an extension step, each at 68 °C for 30 s. Eventually a final extension at 68 °C for 5 min was performed. Positive PCR amplicons were then ligated into a *pEASY*®-Blunt T vector (TransGen Biotech) for transforming *E. coli* DH5a competent cells (Tiangen Biotech) and ten clones of each amplicon were eventually selected for Sanger sequencing (Sangon Biotech).

### Multiple sequences comparison and phylogenetic analyses

Detailed information (GenBank accession nos) of the sequences used in this study is provided in Fig. 1. All of the partial ORF33 and ORF30 nucleotide sequences of EHV-1 which were generated in this study were submitted to GenBank, with the following accession nos: MZ561483-MZ561525 and OM047215-OM047257. Sequences were analyzed by MegAlign software in Lasergene v7.1 and a phylogenic network representation of all target sequences was generated using the maximum-likelihood method based on the Tamura–Nei model of MEGA7. The accuracy of tree topologies was also evaluated using 1000 bootstrap replicates [30].

## Supplementary Information

**Additional file 1: Table S1.** Primer Sequences used in this study.

## Data Availability

All data generated or analyzed during this study are included in this published article and its additional files. Sequences of EHV-1 from aborted fetuses generated in this study have been submitted to GenBank under accession nos: MZ561483-MZ561525 and OM047215-OM047257.

## Abbreviations

EHV: Equid herpesvirus
ORF: Open reading frame
G: Guanidine
A: Adenine
N: Asparagine
D: Aspartic acid
