# Supplementary material for: Outbreak of neuropathogenic equid herpesvirus 1 causing abortions in Yili horses of Zhaosu, North Xinjiang, China
Source: BMC Vet Res. 2022 Mar 1;18:83. doi: 10.1186/s12917-022-03171-1 (PMC8886757; doi:10.1186/s12917-022-03171-1)
Supplement: Supplementary file 1 — Additional file 1: Table S1. Primer Sequences used in this study. [file 12917_2022_3171_MOESM1_ESM.docx]

**Table S1** Primer Sequences used in this study

| Primer | Sequence (5′-3′) | Product size (bp) |
| --- | --- | --- |
| EHV-4 ORF33(gB)-F | TGGGTCCTACAGATTTACTATTCG | 587 |
| EHV-4 ORF33(gB)-R | TACACCACGCAGTTGCTATCCTAC |  |
| EHV-5 ORF8(gB)-F | TGTATCACCGTGGACCAAGAGAG | 881 |
| EHV-5 ORF8(gB)-R | TCAAAGATGGATCTTCTCTGAGTG |  |
| EHV-2 ORF8(gB)-F | GGTGACACTATAGAGATGTCRCC | 716  592  559 |
| EHV-2 ORF8(gB)-R  EHV-1 ORF33(gB)-F  EHV-1 ORF33(gB)-R  EHV-1 ORF30(viral DNA polymerase)-F  EHV-1 ORF30(viral DNA polymerase)-R | CTGTTGATGCTCTTTCTGAGATTG  GGGTCCTACAGATTTACTATTCG  TACACCACGCAGTTGCTATTCTAC  CGGAGTAAGGCTTGTGGTTTCG  GTGGGCTACCAGGGAGCAAAG |  |
